# Parents’ and caregivers’ experiences and behaviours when eating out with children with a food hypersensitivity

**DOI:** 10.1186/s12889-017-4594-z

**Published:** 2017-07-20

**Authors:** Fiona M. Begen, Julie Barnett, Miriam Barber, Ros Payne, M. Hazel Gowland, Jane S. Lucas

**Affiliations:** 1 Department of Psychology, University of Bath, Claverton Down, Bath, UK; 2 Creative Research Ltd, Bishops Castle, UK; 3 Allergy Action, St Albans, UK; 4 Clinical & Experimental Sciences, Faculty of Medicine, University of Southampton, Southampton, UK

**Keywords:** Food allergy, Food intolerance, Food hypersensitivity, Allergen avoidance, Eating out, Children, Parents

## Abstract

**Background:**

For parents and caregivers of food hypersensitive (FH) children, accommodating their child’s dietary needs when eating out can be a challenging experience. This study explored caregivers’ experiences and behaviours when eating out with their FH child in order to gain insights into how they support and prepare their child in negotiating safe eating out experiences.

**Methods:**

A cross-sectional, qualitative design was used. In depth, semi-structured interviews were carried out with 15 caregivers of children with FH. Interviews were analysed using framework analysis.

**Results:**

Caregivers reported a number of issues relating to eating out with their FH child, or allowing their child to eat out without their supervision. Through themes of ‘family context’, ‘child-focused concerns’, and ‘venue issues’, caregivers described how they managed these and explained the limitations and sacrifices that FH imposed on their child, themselves, and family members.

**Conclusions:**

Through deeper understanding of the anxieties, negotiations and compromises experienced by caregivers of children with FH when they are eating out, clinicians and support charities can tailor their support to meet the needs of caregivers and children. Support and education provision should focus on providing caregivers of children with FH the tools and strategies to help enable safe eating out experiences.

**Electronic supplementary material:**

The online version of this article (doi:10.1186/s12889-017-4594-z) contains supplementary material, which is available to authorized users.

## Background

There is no cure for food hypersensitivity and management is restricted to avoidance of the implicated food and treatment of symptoms caused by accidental ingestion. Food hypersensitivity (FH) can impact adversely on the child and family with regards to daily living and quality of life [1], as well as on nutrition and wellbeing. By the age of 3 years, 6% of children suffer from FH confirmed by food challenges; however a much larger proportion of caregivers report food-related symptoms that lead them to avoid particular foods [2].

Food hypersensitivity is a generic term, used for individuals who suffer reproducible, objective symptoms whenever they eat a particular food [3]. FH includes food allergy and non-allergic food hypersensitivity. Food allergies are caused by abnormal immunological responses to a food, whereas food intolerances have a non-immunological basis. Food allergies are usually mediated by IgE; reactions typically occur extremely quickly after ingestion and although symptoms are sometimes mild (e.g. urticarial rash or itchy mouth), reactions can be life-threatening (anaphylaxis) [4, 5]. People with food allergies need to be ready to treat reactions caused by accidental ingestions with oral antihistamines for milder symptoms, or injectable adrenaline (epinephrine) for anaphylaxis. Non-allergic food hypersensitivity is a heterogeneous group of conditions without an identifiable immunological basis (e.g. lactose intolerance). Food intolerances generally have a delayed reaction, and rarely have immediate clinically severe consequences, although they can cause severe chronic disease such as malnutrition. Coeliac disease is an autoimmune disease which causes gluten intolerance; it can be classified as a non-IgE mediated allergy. Non-IgE mediated allergy and non-allergic hypersensitivity share features of delayed onset of reactions, extremely low risk of immediate life threatening complications and lack of effective treatments for symptoms; for the purpose of this manuscript we have therefore combined non-IgE mediated allergy and non-allergic hypersensitivity as ‘food intolerant’. We use the term food hypersensitivity to include both food allergy and food intolerance.

What unifies people with FH is the need to avoid food allergen(s) in order to reduce symptoms, for long-term health implications, or healthy days lost due to adverse reactions [6]**.** For caregivers of children with a FH, allergen avoidance requires constant vigilance and represents an ongoing day-to-day challenge [7]. Broadly, the experience of being a caregiver to a child with FH has been described as ‘living with risk’ [8]. This is typified by higher levels of anxiety [9], distress and worry, and a need for greater negotiation and compromise during family activities [1, 10, 11]. The measures taken to avoid allergens will depend on a number of factors including previous severity of reactions and advice from clinicians; for example, there will be significant differences in self-management responsibility for caregivers of a peanut allergic child at risk of anaphylaxis versus a child that is lactose intolerant.

For caregivers of children with FH, accommodating the child’s dietary needs when eating out poses unique considerations and challenges. The risk of cross contamination of food products; the necessity to entrust to others the care or partial care of the child; and the adequacy of labelling; may all have the potential to breach or threaten safe practices instituted in the home environment [7]. Appreciating how caregivers approach, cope and manage these specific instances constitutes an important and novel line of inquiry, with practical applications that can inform clinicians and the healthcare sector in providing education and support for both children with FH and their caregivers.

The study aimed to understand caregiver’s experiences and behaviours when eating out with their child, and how they support and prepare their child in negotiating safe eating out experiences. The research is the first to purposefully investigate the eating out experiences, behaviours and concerns of caregivers of children who they perceive to be at risk of adverse reactions to particular foods. We aimed to include caregivers of children reporting a range of symptoms associated with food allergy and food intolerances. By including participants who were self-diagnosed alongside those who were under specialist allergy services, we aimed to capture a variety of experiences, severity of reactions, risks of reactions and levels of knowledge.

## Methods

This was a qualitative interview study conducted with caregivers of children with food hypersensitivity.

### Recruitment and population

Ethical approval was gained from the University of Bath, Department of Psychology Ethics Committee (Ethical Approval Ref: 14–055). A specialist market research agency (Acumen Fieldwork- Medical) recruited 75 FH participants from across the UK [12] using purposive sampling. Key inclusion criteria were that participants reported (a) having a reaction to food, and (b) avoiding one of the 14 allergens^1^ that were the focus of Europe-wide EU legislation introduced in December 2014 [13]. This required providers of non-prepacked foods to provide written and verbal information related to the content of these allergens within their foods. From this larger population (which included adults with FH themselves), 15 caregivers of children aged less than 18 years that had FH completed in-depth interviews (14 of these the mothers of the children). Interviews were conducted between June and August 2014. Prior to interview, caregivers completed a screening questionnaire characterising their child’s reactions to one or more of the 14 specified allergens in order to ensure that the sample included children with a range of reactions to a range of allergens. A breakdown of children’s characteristics is provided in Table 1. The majority of participants (*n* = 12, 80%) had received a formal diagnosis from a health professional or hospital clinic. Of these formally diagnosed participants, caregivers specified the clinical diagnosis that their child had received as: food allergy only (*n* = 5, 42%), food allergy and food intolerance (*n* = 4, 33%) (for example, food allergy to peanuts and food intolerance to cereals containing gluten), food intolerance only (*n* = 2, 17%) (1 with milk and/or lactose intolerance only; 1 with milk and/or lactose intolerance and at least one other intolerance), and coeliac disease only (*n* = 1, 8%). Three participants were self-diagnosed with food intolerance by caregivers. Caregiver demographic details are available in Additional file 1: Table S1.

**Table 1:** Characteristics of the 15 food hypersensitive children as reported by their caregivers

Variable	Total (%) *N* = 15
Gender	
Male	8 (53.3)
Female	7 (46.7)
Age group (years)	
< 8	4 (26.7)
8–12	3 (20.0)
13–17	8 (53.3)
Diagnosis	
Clinical diagnosis * (by GP; Dietician or Allergy specialist at hospital)*	12 (80.0)
Self-diagnosis * (by caregiver)*	3 (20.0)
Time since diagnosis (years)	
1–5	10 (66.7)
> 5	5 (33.3)
Self-reported timing of reactions	
Reaction starts immediately or within the hour	10 (66.7)
Reaction starts 1–24 h later	5 (33.3)
Nature of worst self-reported reactions	
Generally associated with IgE-mediated reactions (*Includes at least one of the following symptoms: ‘Stinging nettle’ rash, urticaria, hives, itching or swelling of the lips, tongue or mouth, asthma, wheezing, facial swelling, breathing difficulties, anaphylaxis, collapse. May additionally include symptoms associated with non-IgE-mediated reactions*)	9 (60.0)
Generally associated with non-IgE-mediated reactions (*Includes at least one of the following symptoms: vomiting, diarrhoea, sneezing, catarrh, hyperactivity, tiredness, stomach cramps, other digestive problems* (*e.g. bloating, constipation), eczema flare, migraines/headaches, aching joints/muscles, behavioural/mood changes; but does not include symptoms associated with IgE-mediated reactions*)	6 (40.0)
Allergens	
Peanut	6 (40.0)
Tree nut	5 (33.3)
Sesame	1 (6.7)
Cereals containing gluten	2 (13.3)
Milk	9 (60.0)
Crustaceans	1 (6.7)
Eggs	1 (6.7)
Soya	1 (6.7)
Multiple allergens (≥2)	7 (46.7)
Treatment	
Allergen avoidance	15 (100.0)
Antihistamines	10 (66.7)
Injectable adrenaline	3 (20.0)
Inhaler	2 (13.3)
Special diet *(in addition to allergen avoidance)*	5 (33.3)
Multiple treatments (≥2)	4 (26.7)

### Procedure

In-depth interviews were carried out with participants in their own homes. Interviews lasted between 38 min-1 hr 43 min (mean 1 hr 7 min) and were recorded with permission of the participant. Initial questions engaged participants with the topic of food and their/their child’s experiences relating to of having FH, their adaptation to this, and day-to-day coping strategies. The interview then focused on participants’ experiences and behaviours when eating out with their FH child. Participants were encouraged to discuss the implications of their child’s FH within the context of eating out and to elaborate on their child’s and their own responses in negotiating a satisfactory eating out experience. The development of the interview schedule was informed by a literature review, discussions with support groups and the particular expertise and experiences of two of the study authors: JSL is a Professor of Paediatric Respiratory Medicine and MHG who has a severe peanut and tree nut allergy.

### Analyses

Interview recordings were fully transcribed and any ambiguities in transcription checked against the recordings. The research questions were addressed using framework analysis [14]. Framework analysis has become popular in social, policy, and health research because it applies a systematic approach which prioritises the transparency of the analytical process; thereby maximising accessibility and strengthening confidence in subsequent results and conclusions [15, 16]. Interviews were coded and analysed by FMB using the qualitative analysis software QSR NVivo (version 10). Further analysis and the development and discussion of themes was conducted in regular meetings with JB. Themes were subsequently reviewed and results discussed with RP, JSL and MHG.

Identified themes are illustrated with quotes. Where quotes are used, participant details are indicated in brackets as follows: Participant number; Child’s gender (M/F), age group (<8, 8–12, 13–17) and reported food allergens. Italicised text reflects prompts used by the interviewer.

## Results

Three overall themes were described by caregivers in relation to their eating out experiences with a FH child. Responses focused on eating out and management of FH in relation to: ‘family context’, ‘child-focused concerns’, and ‘venue issues’.

### The family context

Participants emphasised the importance of eating out as a family. For many caregivers, eating out together served as a means of cementing family relationships; allowing dedicated time to catch up with their children’s lives, away from the day-to-day stresses of work (Table 2: Quotes 1 & 2).

**Table 2:** The family context of eating out with a child with FH

Importance of eating out for family:
1) Yes, we’re going out this Saturday, my husband’s from (UK city), and his mum’s from (UK city), her brother is from (European country), with his wee girl., and we’re all going out as a family, which is really nice.…We always try and go out, we were out last Friday at the (chain restaurant), because my husband and I both work, we like to go out, spend family time together, and catch up with (daughter). It’s very important. (P6, F 8–12: Peanuts & tree nuts)
2) (Burger chain drive-through) the one Friday of the week. That’s because that’s mine and his thing together. That’s me finished work… so that’s the wee snippet of time that I have and he just thinks it’s great fun in the car. (P9, M < 8: Milk)
Impact on other family member’s eating out behaviours and freedom of choice:
3) *So how often would you say you are eating out?*
It could be once a week, it used to be more, now of course it’s a bit less, it could be once a week, yeah. * So his condition has sort of reduced the frequency which you’d do that?* Yeah because the variety of places you can go and you know he’ll be okay are less now. * Was that just you know whenever you felt like it or would you tend to go and eat out on a special occasion?* Special occasions and sometimes like at the weekend, I’ll just think ‘oh I’m not cooking, let’s just go out’. But you have to think a bit more because if there are only say one or two things that we know are safe for him on the menu….So it’s kind of restricted our choice of places to go. (P1, M 13–17: Peanuts, tree nuts, milk)
4) The (restaurant name), that is my husband’s favourite (Asian restaurant), but we don’t go so much now because it’s very difficult for him (son) to find some curry and rice, to find you know what you call a ‘meal’ that would be safe for him. (P2, M 8–12: Peanuts, tree nuts)
5) We…have a bit of a chat about it because Dad will say to (child’s sibling) ‘don’t be ridiculous, we’re not going to (chicken chain restaurant)’, Mum and (daughter) need something else. I mean we’ve been out before and we’ve split up to different places for lunch….Which can get irritating. (P5, F 13–17: Cereals containing gluten)

Caregivers described the challenges and compromises associated with ensuring a safe eating out experience for their FH child, whilst also satisfying the preferences of other family members. Both the frequency of eating out and venue selection were affected by the child’s FH (Table 2: Quote 3). In some instances this led to family members foregoing their preferences in favour of a ‘safer’ venue for the child (Table 2: Quotes 4 & 5). There were also occasions in which the FH child was seen to compromise in order to ensure that their siblings were given the opportunity to eat out at venues that they enjoyed. The balance between finding safe and appropriate venues, whilst simultaneously trying to meet the preferences of family members represented an inherent problem for caregivers, given that eating out was felt to invariably involve compromise or sacrifice for at least one family member.

### Child focused concerns

Caregivers were concerned by the restrictions that FH placed on their child when eating out. They empathised with their child’s frustration and disappointment, and expressed sensitivity towards feelings of *difference* and *alienation*, borne out of limited food choices and the propensity for children to make comparisons to the eating out experiences of unaffected peers (Table 3: Quotes 1 & 2).

**Table 3:** Child-focused concerns when eating out

Concerns about limitations on child eating out:
1) Yeah, I think it’s embarrassing (for child), because he doesn’t want to feel different, nobody wants to feel different. If someone else had the problem he wouldn’t feel so bad but because he’s the only one out of us, yeah I mean he does feel different. (P8, M 13–17: Peanuts, tree nuts)
2) I feel it’s quite unfair on him, eating the same thing. He either gets sausages and chips or chicken nuggets or he’ll have plain noodles. It’s not really fair....it’s not fair on him when every other child gets an ice cream and he gets a packet of crisps. Every other child gets chocolate and he gets a bowl of grapes. (P9, M < 8: Milk)
Avoiding child’s disappointment:
3) Yeah it impacts a lot because most desserts, unless he just gets fruit, he can’t have them. So what I do then is if he’s made something or I’ll try and get something he can have when we go back home. (P1, M 13–17: Peanuts, tree nuts, milk)
4) We tend to stick to the same place if you’ve been there once and its ok, and if he doesn’t have a reaction…if he likes it, it’s a bonus. (P2, M 8–12: Peanuts, tree nuts)
5) ….there was this one time I took him to a vegan restaurant and he was just in heaven because they had the most incredible puddings. We’ve got to go back there but it’s just not very near. If only there were more vegan restaurants! (P13, M 13–17: Milk)

Caregivers believed that their child was ‘missing out’, and endeavoured to compensate for this where possible (Table 3: Quote 3). They emphasised ways in which safety was supported and negative experiences were mitigated. Caregivers favoured familiar eating out venues which had a proven track-record for providing allergen-free dishes, which the child had enjoyed on previous occasions (Table 3: Quote 4). Venues that were guaranteed as allergen free were a source of elation for the child (Table 3: Quote 5); and for caregivers, were a welcome contrast to venues where choices were limited (cross reference to Table 3: Quotes 1 & 2).

Caregivers described the age-related characteristics and issues encountered by their FH child when eating out. Caregivers of younger children assumed the main responsibility in avoiding allergens. However this was seen to be tested by the curiosity and temptation that young children - in particular - could show towards other people’s food (Table 4: Quote 1).

**Table 4:** Age-related factors regarding allergen avoidance when eating out

1) I just know he’ll be sick so I’m quite strict about that. But he is starting to notice what other children are having and what his big brother’s having and getting a bit upset that he can’t get it. So that’s only going to get worse. (P15, M < 8: Peanuts, tree nuts, eggs, milk, cereals containing gluten)
2) (Caregiver addressing child) You were going every Saturday with your school friends…..And there’s a group of boys and girls and boys are always hungry. But you always have to just have a cup of tea don’t you….You end up at (burger chain). You just eat chips, it’s not a meal though, and it’s just having something to eat. (P5, F 13–17: Cereals containing gluten)
3) If the boys had a party obviously they could go, but if it was (daughter) she would go just to support them, but she wouldn’t be able to eat anything there……Because I couldn’t tell her friends ‘Oh you can’t go to (pizza chain) for your birthday because my daughter is allergic to cheese’. So she’d go along and sit along. (P11, F 13–17: Milk)
4) He’d gone out with (his sister), she was being a bit dithery, they both had some chocolate. Without her thinking, he wanted to try something of what she had. Because she’d already started eating it, she said oh all right try a bit then. Not paying attention, she (hadn’t) realised there was some nuts in there. Until he started saying, (sister’s name)…. he’s quite competent in knowing when, or piping up and saying there’s something not quite right here.
5) I think she’ll continue to be good with it, because she’s had to get used to it from such a young age that she doesn’t know any different; she’s got it. You do get the odd times where she’s said’ I’d love to have nuts, I’d love to have them’. (P6, F 8–12: Peanuts, tree nuts)

Caregivers indicated that with development, the division of responsibilities in managing the child’s food hypersensitivity were renegotiated. Caregivers of older children suggested that with age, children’s awareness towards their food hypersensitivity and what this means in the context of their social lives, becomes more apparent. Socialising with friends whilst accepting the restrictions of allergen-avoidance when eating out was felt to be particularly problematic for older children (Table 4: Quotes 2 & 3). Caregivers empathised with the complexities of this period. However owing to the increasing autonomy of children at this stage of development, caregivers showed awareness about changes in the extent of their own influence, and recognised the need for increased confidence in the child’s self-management (Table 4: Quotes 4 & 5).

Caregivers reported using a number of strategies to assist their child in managing their FH when eating out. From a young age, children were encouraged to be knowledgeable about ‘safe’ food options and the role of exercising caution (Table 5: Quote 4). Caregivers were keen to foster independence in their children, and for them to be informed about susceptible food allergen(s) (Table 5: Quotes 1–3). Caregivers also endeavoured to equip children with self-assertion skills, so that they could effectively make others aware of their food hypersensitivity. In all instances, caregivers expressed the value in this becoming a habitual approach for their children to assume, and pointed to their own role in supporting the development of this (Table 5: Quotes 2 & 3).

**Table 5:** Guiding child’s choices, managing their anxieties, and supporting their autonomy when eating out

Assisting child in making safe choices when eating out:
1) …there are times where we’ve stayed in hotels and had breakfast in the morning and again, she’s fine, just go with her but yeah, it’s been fine. (P4, F < 8: Milk)
2) I’m trying to get her into the habit of…asking (in school canteen)…It’s a limited window of time, it’s a case of getting in, sitting down and get eating, but I’ve said to her to ask the ladies behind the desk, just check with them, She needs to get into the habit, next year she’ll be in year 5, her confidence is increasing. (P6, F 8–12: Peanuts, tree nuts)
3) I’ve just drummed it into him wherever he goes… and if he’s eating out, just say allergic to peanuts or nuts, and stay away from them. It’s different if I’m there. (P12, M 8–12: Peanuts, milk)
4) He knows he can go somewhere and pick up something he knows is safe and he’s not feeling self-conscious at all but I think he would be awkward if he wanted to ask. But he’s going to have to do it. (P8, M 13–17: Peanuts, tree nuts)
Caregiver’s trust in child’s confidence eating out:
5) I mean he is sensible. He knows what he can and can’t have, and his friends know. They’ll go to (burger chain) and he’ll have chips and an ice cream because he knows he’s fine with that…..His friends accept it; they’ve known him since primary school. It’s not a new concept with them so he’s fine with that, he just knows what he can and can’t have. (P8, M 13–17: Peanuts, tree nuts)
6) …my son just goes on the internet to see what’s in this, what is in that. He does that a lot. * Right so he’s really sort of tried to take control.* Very much so, he’s completely taken control of his own situation, so that helps me a lot. (P1, M 13–17: Peanuts, tree nuts, milk)
7) She doesn’t see it as a problem. So I myself, her dad lives in (European country), by the seaside and there’s lots of places to eat by the seafront and I would avoid those places. (Child responding) Really? When I went to (European country) without you, we went. (P10, F 13–17: Crustaceans)
8) …*is there anything you ever do that sometimes makes you feel anxious?* (Caregiver addressing child) You’re not bothered, are you? It doesn’t seem to affect him. It’s more us, definitely. (P12, M 8–12: Peanuts, milk)
Balancing the need for caution with avoidance of worry and anxiety when eating out:
9) …since the last time it happened, she’s very, very, even since she’s been sick bless her, she’s been so panicky about them. I mean she’s obviously at home, she knows that I’m on top of it but yeah, if we do go out… if there’s something slightly or something that looks like a sesame (seed), you know literally anything, she’ll be very… ‘are you sure, are you sure, are you sure?’ (P3, F < 8: Sesame)
10) (When trying new food) it would be taking a little bit at a time…and seeing what his reaction would be, without trying to freak him out. Like ‘Oh you can only have a spoonful of that, then wait for 5 min!’ So it’s almost about being a little deceptive about how you do things. (P2, M 8–12: Peanuts, tree nuts)
11) He knows the food he can eat and he tends to stick with that… He should try a bit more variety but he won’t….We’ve tried everything to get him to eat something a bit different… (P12, M 8–12: Peanuts, milk)

Some caregivers were reassured by their older child’s confidence in selecting safe options when eating out (Table 5: Quotes 5 & 6). However, others were concerned that their child’s confidence might be misplaced or lead to complacency. This represented a disparity in outlook between the caregiver and child (Table 5: Quotes 7 & 8).

Caregivers described the complexities of trying to balance the need for caution with the need for variety in eating out experiences. For some children, the need to exercise caution led them to be hesitant and over precautionary in their food choices; even in the face of guidance from the caregiver (Table 5: Quotes 9–11). Caregivers who noticed children assuming a precautionary approach at the expense of safe exploration, described their efforts to support the child in negotiating the balance between safety and food variety (Table 5: Quotes 10 & 11). When negotiation around these considerations was unsuccessful, this was met with regret that their child was losing out on culinary variety (Table 5: Quote 11).

### Venue focused issues

Although caregivers favoured frequenting familiar eating out venues, where this was not possible a process of ‘trial and error’ was used to explore viable eating out options. They reported being thorough and taking the necessary time to seek information from menus and venue staff; whilst accepting the inherent risk that was involved when eating out in unfamiliar environments (Table 6: Quotes 1–3).

**Table 6:** Managing concerns relating to eating out venues and concerns surrounding allowing child to eat out independently

Trial and error when eating out:
1) Initially when we found out about (son’s) allergy, we had to tentatively go into these places, we had to scour the menus, and ask questions of the staff,…and being conscientious about trying something…but, because it’s (Asian restaurant) one of these places where you can eat as much as you like, it would be taking a little bit at a time… (P2, M 8–12: Peanuts, tree nuts)
2) Sometimes you will buy something you know and if you go to a deli or something and things are just a bit loose and you can sort of pick and mix, you can try to think what you think would be best and what would be safe, but sometimes you just honestly don’t know exactly what is in something. So that makes it sometimes a bit risky and sometimes you just trial it once. (P1, M 13–17: Peanuts, tree nuts, milk)
3) (Child response) If it’s (burger chain) chips or something like that then I’ll take a bit of a risk, just to see if I can actually eat there. And if it hurts, I’ll just leave it. You just have to or else you won’t eat. (P5, F 13–17: Cereals containing gluten)
Relying on others to ensure child’s safety eating out / away from home:
4) He’s starting the nursery in August….My elder son^a^ went to that nursery so I’m kind of feeling quite confident but there’s all the additional information that I’m going to have to give them about (name of child with FH)….And my friend’s son uses the nursery and they said they’ve got photos of the children and what they can and cannot eat so they know what their allergies are. So it’s taken very seriously. And certainly the crèche has been fantastic. (P15, M < 8: Peanuts, tree nuts, eggs, milk, cereals containing gluten)
5) ….even if it’s to do with the canteen and actually the chefs and stuff within the school. They send a letter home – … you have to fill out a general form – I think it might just be at the beginning of every year just for the kids that are likely to have school meals and it does say straight away any allergies you have to specify them there. (P3, F < 8: Sesame)
6) At the start (nursery) struggled….They have had a couple of slip ups like the (ready mix dessert) and a couple of times because the teacher will go on holiday….They have had the repercussions….either the projectile vomiting or they have had to change really bad nappies. They don’t hide it, they do tell you and then they apologise profusely…I feel quite let down sometimes because I stress to them when I go in. (P9, M < 8: Milk)
7) (Caregiver addressing child) It might have been when you went back (to school), sort of September time….Well you’d had 6 weeks at home….Then you go back to school, eat something in the canteen….And then you were poorly…. I have complained at school because nowhere else will listen to me…I’ve complained a lot at school about it and they still haven’t done anything. (P5, F 13–17: Cereals containing gluten)
8) Going away on school trips, people not appreciating, my son is fine with this, he won’t have a problem. They don’t appreciate how difficult it actually is and how cautious you have to be. So we had quite a battle with the school regarding that. They wouldn’t sort of appreciate that someone eating peanut butter beside him could cause a problem. But they weren’t willing to put a ban out….. (P8, M 13–17: Peanuts, tree nuts)

^a^Elder son was not food hypersensitive

Particular concerns were raised in the context of young children with FH eating out in nursery/school contexts where food was provided by the nursery/school. Although some caregivers were reassured by experiences of good practice in accommodating their child’s FH (Table 6: Quotes 4 & 5), others reported mistakes which had resulted in their child suffering avoidable symptoms (Table 6: Quote 6). Although these mistakes were often rectified, caregivers expressed concern where there appeared to be a fundamental lack of understanding regarding FH and the consequences for their child (Table 6: Quote 7). In a school setting, this led some caregivers to limit their FH child to food providing from home in the form of ‘packed lunches’. However, many caregivers appreciated the need to take part in everyday social activities, such as eating in the school canteen or going on trips. They did not wish to deprive their child of this experience, and endeavoured to inform and change school practice in order to reduce risk (Table 6: Quotes 7 & 8).

## Discussion

This study provides detailed insights into the experiences and behaviours of caregivers when eating out with their FH child, or allowing their child to eat out without supervision. Through the 3 themes of ‘family context’, ‘child-focused concerns’, and ‘venue issues’, caregivers revealed the limitations that FH imposed on their child, themselves as caregivers, and wider family, as well as the processes taken to minimise risk and enhance safety.

Caregivers described emotional, practical and social factors associated with eating out with their FH child. Many negotiations were made with respect to balancing the need for: caution with variety; safety with social freedom; and preferences of the FH child with preferences of siblings. We recognise that to some extent caregivers of children who do not have a food hypersensitivity may experience attenuated versions of these dilemmas when eating out. Caregivers of children with food hypersensitivities described the steps taken to strike these balances, including compromise and positive risk taking. These align with those detailed in the theoretical framework proposed by Broome et al. [7]. This describes caregivers of children with hypersensitivities traversing a three-phase process: questioning competency (the goal of the stage is to secure support), expanding competency (the goal of the stage is to minimise risk and provide a safe environment) and regaining competency (the goal of the stage is to establish a ‘new normal’) [7].

Overall caregivers assumed the responsibility in managing their children’s food hypersensitivity. This was particularly evident in caregivers of young children compared to the recognition and somewhat tentative encouragement of the ability of older children to make safe food choices. Caregivers also used a number of strategies to support self-management and autonomy in their children. Children were encouraged to clearly declare their dietary needs, and it was evident that some older children were utilising these techniques effectively in adolescence; which reassured some caregivers. These strategies were built on the understanding that children would be operating in contexts without caregivers being present, and reflected a shift in responsibility over the course of development.

In several instances, difficulties associated with eating out related to a lack of understanding of FH on the part of eating out venues. In particular, and in line with other literature, there were concerns from caregivers in instances when they were not present themselves e.g., in nurseries and schools [17, 18]; though favourable experiences and outlooks in the context of such venues were also acknowledged. In providing a backdrop to the concerns, it was apparent that caregivers experienced their child’s FH from the perspective that they, as caregivers, bore primary responsibility for their child’s food allergen avoidance; regardless of eating out context. This responsibility represented an additional burden and source of distress for the majority, and caregiver’s experienced relief in venues that could safely cater for the FH child.

In capturing the variety of experiences, severity of reactions, risks of reactions and levels of knowledge on the part of caregivers of FH children when eating out, it was notable that the core themes of ‘family context’, ‘child-focused concerns’, and ‘venue issues’ were consistently observed amongst this population. There was no evidence that caregivers differed in eating out practices or the vigilance of their allergen avoidance relating to whether the child was food allergic or food intolerant. Although there were only three children that were self-diagnosed there was similarly no evidence of such differences between their caregivers and the caregivers of those whose child had received a formal diagnosis. Surprisingly perhaps, caregivers of children who were at risk of life-threatening anaphylaxis and had been prescribed epinephrine auto-injectors, did not raise this as a management strategy in the context of eating out. Whilst we might have expected these caregivers to be more vigilant in allergen avoidance than other caregivers of FH children; there was no evidence that this was the case. Of the three caregivers whose children had been prescribed auto-injectors, two caregivers mentioned its carriage and use- they hadn’t had to use it but felt confident in its use if needed; and one caregiver did not mention it at all.

### Implications

Many early experiences of eating out take place within a nursery/school setting, and these environments are important in helping to establish constructive approaches and a sense of safety for children with FH and their caregivers. School canteens largely function as private contractors outside mainstream school systems, and may not be equipped to accommodate special diets [19]. There is also the potential for understanding and communication regarding the FH status and management of each child to break down as a consequence of overall school policy [17, 18], and the way some canteens are set up within the school environment. For young children with FH, it is crucial that schools establish mechanisms that protect them from allergen exposure, whilst ensuring that they are not unduly restricted in their food choices [20]. For older children with FH, it is essential that caterers provide accessible, age-appropriate allergen information as a basis for them to make independent food choices. Training staff in order to increase their health literacy around food hypersensitivities [7] alongside coherent ‘management plans’, may also serve to put caregivers at greater ease [1]. There was no evidence in this study that caregivers of children at risk of anaphylaxis saw carrying epinephrine auto-injectors as core to managing the risks of eating out. However, given that eating out is implicated in 50% of deaths related to food allergen consumption [21] this is a key setting where caregivers should be encouraged to ensure that rescue medication is to hand.

Clinicians and FH support charities have important contributions to make by educating and encouraging children with FH and their caregivers to be confident to request the allergen information that eating out venues are now required to provide [12, 22] as a result of Europe-wide EU legislation introduced in December 2014 [13]. Caregivers should be encouraged to use practical techniques, for example: informing eating out venues in advance [5], and supporting their child’s involvement in these responsibilities as befits their developmental stage. Clinicians, support charities and retailers/caterers can further contribute by helping caregivers to construct an individual ‘management plan’ within their child’s nursery/school. Supporting practical ‘FH inclusive’ strategies can limit accidental food allergen exposure, whilst avoiding the feelings of alienation reported by many children with FH [23, 24]. For these children, such strategies could bolster their quality of life in circumstances where this might otherwise be compromised as a result of the stress and anxiety associated with the daily management of the condition [25].

### Limitations and future directions

In recognising the insights gained through the in-depth analysis of caregivers’ experiences when eating out with their FH child, we also acknowledge that sample size was relatively small; though comparable to other qualitative studies within the same field [26–28]. Further qualitative work with a broader selection of caregivers of children with FH would be helpful in establishing the applicability of these findings. Follow-up research is recommended to re-assess caregiver experiences of and behaviours in eating out with an FH child following the implementation and enforcement of the EU legislation requiring enhanced allergen information provision [13].

A further limitation was using caregiver reports of food allergy or intolerance to diagnose FH. We wished to capture families whose children avoided foods because of perceived reactions. By including participants who were self-diagnosed alongside those who were under specialist allergy services, we captured a variety of experiences, severity of reactions, risks of reactions and levels of knowledge. The prevalence of FH is considerably greater when diagnosis is captured by caregiver reports rather than ‘gold-standard’ double-blind placebo controlled food challenges [2, 29] but for the purposes of this study it was important to capture the experiences of all families who perceived a risk of reacting to allergens.

## Conclusions

This qualitative study gained insights into caregivers’ experiences and behaviours when eating out with their FH child, and how they supported and prepared their FH child in negotiating safe eating out experiences. Through the themes of ‘family context’, ‘child-focused concerns’, and ‘venue issues’, caregivers described the constraints and sacrifices that FH imposed on their child, themselves as caregivers, and wider family. Through deeper understanding of the subtle anxieties, negotiations and compromises experienced by caregivers of children with FH when they eat out, clinicians and support charities, and retailers can tailor their support and information provision to meet the needs of caregivers and children. Education should focus on providing both caregivers and eating out venues with the tools and strategies to ensure positive eating out experiences for children with FH. Cumulatively, this has the potential to reduce the day-to-day anxieties of caregivers and to provide them and their children with improved quality of life.

## Additional file

Additional file 1: Table S1. Characteristics of the 15 caregivers of food hypersensitive children. Description of data: Characteristics of 15 caregivers of food hypersensitive children. (DOCX 29 kb)

## Abbreviations

FH: Food hypersensitivity

## Footnotes

1 Peanuts, tree nuts, cows’ milk, soya, mustard, lupin, hens’ eggs, fish, molluscs, crustaceans, cereals containing gluten, sesame seeds, celery and sulphur dioxide (at levels above 10 mg/kg, or 10 mg/l).
