# Supplementary material for: Parents’ and caregivers’ experiences and behaviours when eating out with children with a food hypersensitivity
Source: BMC Public Health. 2017 Jul 20;18:38. doi: 10.1186/s12889-017-4594-z (PMC5520367; doi:10.1186/s12889-017-4594-z)
Supplement: Additional file 1: Table S1. — Characteristics of the 15 caregivers of food hypersensitive children. Description of data: Characteristics of 15 caregivers of food hypersensitive children. (DOCX 29 kb) [file 12889_2017_4594_MOESM1_ESM.docx]

Additional file information-

File name: Additional file 1

File format: docx

Title of data: Additional file 1: Characteristics of the 15 caregivers of food hypersensitive children

Description of data: Characteristics of 15 caregivers of food hypersensitive children

Additional file 1: Characteristics of the 15 caregivers of food hypersensitive children

| Variable | Total (%)  N=15 |
| --- | --- |
| Gender |  |
| Male | 1 (6.7) |
| Female | 14 (93.3) |
| Age group (yrs)^*^ |  |
| 18-30 | 2 (13.3) |
| 31-45 | 5 (33.3) |
| 46-60 | 7 (46.7) |
| UK country of residence |  |
| England | 7 (46.7) |
| Northern Ireland | 2 (13.3) |
| Scotland | 4 (26.7) |
| Wales | 2 (13.3) |
| Ethnicity |  |
| White British | 13 (86.6) |
| Caribbean | 1 (6.7) |
| Asian | 1 (6.7) |
| Occupation^*^ |  |
| Intermediate managerial, administrative/professional | 2 (13.3) |
| Supervisory/clerical and junior managerial,  administrative/professional | 5 (33.3) |
| Skilled manual | 5 (33.3) |
| Semi-skilled/unskilled manual | 1 (6.7) |
| Not working | 1 (6.7) |

^*^ One missing value
